# Huashi baidu granule in the treatment of pediatric patients with mild coronavirus disease 2019: A single-center, open-label, parallel-group randomized controlled clinical trial

**DOI:** 10.3389/fphar.2023.1092748

**Published:** 2023-01-19

**Authors:** Jiande Chen, Qiuyu Tang, Baoqin Zhang, Shuhua Yuan, Jia Chen, Shiyu Shen, Dong Wang, Jilei Lin, Hongliang Dong, Yong Yin, Jian Gao

**Affiliations:** ^1^ Department of Respiratory Medicine, Shanghai Children’s Medical Center, School of Medicine, Shanghai Jiao Tong University, Shanghai, China; ^2^ Department of Respiration, Fujian Branch of Shanghai Children’s Medical Centre Affiliated to Shanghai Jiao Tong University School of Medicine (Fujian Children’s Hospital, Fujian Maternity and Child Health Hospital, College of Clinical Medicine for Obstetrics and Gynecology and Pediatrics, Fujian Medical University), Fuzhou, Fujian, China; ^3^ Department of Paediatrics, Taicang Affiliated Hospital of Soochow University, The First People’s Hospital of Taicang, Jiangsu, China; ^4^ Department of Traditional Chinese Medicine, Shanghai Children’s Medical Center, School of Medicine, Shanghai Jiao Tong University, Shanghai, China; ^5^ Infection Department, Fujian Branch of Shanghai Children’s Medical Centre Affiliated to Shanghai Jiao Tong University School of Medicine (Fujian Children’s Hospital, Fujian Maternity and Child Health Hospital, College of Clinical Medicine for Obstetrics and Gynecology and Pediatrics, Fujian Medical University), Fuzhou, Fujian, China; ^6^ Pediatric Translational Medicine Institute, Shanghai Children’s Medical Center, School of Medicine, Shanghai Jiao Tong University, Shanghai, China

**Keywords:** COVID-19, SARS-CoV-2, omicron, traditional Chinese medicine, clinical trial, number NCT05501288

## Abstract

**Background:** Since late February 2022, a wave of coronavirus disease 2019 (COVID-19) mainly caused by the severe acute respiratory syndrome coronavirus 2 (SARS-CoV-2) Omicron variant rapidly appeared in Shanghai, China. Traditional Chinese medicine treatment is recommended for pediatric patients; however, the safety and efficacy remain to be confirmed. We conducted a single-center, open-label, parallel-group randomized controlled trial to assess the efficacy and safety of a Chinese herb compound, Huashi Baidu granule (HSBDG) in pediatric patients with laboratory-confirmed mild COVID-19.

**Methods:** 108 recruited children (aged 3–18 years) with laboratory-confirmed mild COVID-19 were randomly allocated 2:1 to receive oral HSBDG for five consecutive days (intervention group) and to receive compound pholcodine oral solution for five consecutive days (control group). The negative conversion time of SARS-CoV-2 nucleic acid and symptom scores were recorded.

**Results:** The median negative conversion time of SARS-CoV-2 nucleic acid was significantly shorter in the intervention group than in the control group (median days [interquartile range (IQR)]: 3 [3–5] vs. 5 [3–6]; *p* = 0.047). The median total symptom score on day 3 was significantly lower in the intervention group than in the control group (median total symptom score [IQR]: 1 [0–2] vs. 2 [0–3]; *p* = 0.036). There was no significant differences in the frequency of antibiotic use and side effects between the two groups.

**Conclusion:** HSBDG is a safe, effective oral Chinese herbal compound granule, which shows a good performance within the Omicron wave among pediatric patients.

## 1 Introduction

Since late February 2022, a wave of coronavirus disease 2019 (COVID-19) mainly caused by the severe acute respiratory syndrome coronavirus 2 (SARS-CoV-2) BA.2.2 sublineage of the Omicron variant rapidly appeared in Shanghai, China (Zhang et al., 2022). Omicron tends to spread more rapidly but has less virulence than earlier variants (Hui et al., 2022; Suzuki et al., 2022). The predominant clinical manifestations of Omicron have been reported to be upper respiratory infection symptoms such as a sore throat and cough (Kim et al., 2022; Menni et al., 2022). During this Shanghai omicron wave, over 12,000 children have been infected with SARS-CoV-2 and most of the cases were under mild or moderate conditions (Zhou et al., 2022).

According to the latest version of the Chinese diagnosis and treatment protocol for COVID-19 (trial version 9), PF-07321332/Paxlovid and BRII-196/BRII-198 are recommended for adults and adolescents of mild or moderate COVID-19 who have high-risk factors for progressing to severe COVID-19 (National Health Commission of the People’s Republic of China National Administration of Traditional Chinese Medicine, 2022). These treatments are not suitable for most children with mild or moderate COVID-19. Therefore, the alternative medicine of Traditional Chinese Medicine (TCM) has been proposed by the government for the prevention and control of the COVID-19 pandemic (Shanghai Municipal Health Commission and Shanghai Municipal Administrator of Traditional Chinese Medicine, 2022). Accumulating evidence has shown that treating COVID-19 with TCM helps improve the cure rate, shorten the course of the disease, delay disease progression, and reduce mortality (Ren et al., 2020; Huang et al., 2021). However, studies on the effects of TCM treatment on pediatric COVID-19 patients are limited.

Huashi Baidu granule (HSBDG) which is a compound granule composed of 14 Chinese herbs has been developed for clinical application in the treatment of COVID-19 (Liu et al., 2021) and is recommended by the Chinese diagnosis and treatment protocol for COVID-19 (trial version 9) (National Health Commission of the People’s Republic of China National Administration of Traditional Chinese Medicine, 2022). In adult patients, HSBDG combined with standard care for general type COVID-19 could safely improve symptoms but not the time for SARS-CoV-2 nucleic acid negative conversion (Liu et al., 2021). However, the efficacy and safety of HSBDG are unknown in pediatric patients. We conducted a single-center, open-label, parallel-group randomized controlled trial to assess the efficacy and safety of HSBDG in pediatric patients with laboratory-confirmed mild COVID-19.

## 2 Materials and methods

This was a single-center, open-label, parallel-group randomized controlled clinical trial, with an allocation ratio of 2:1. Written consent was obtained from the parents of all participants. The study was conducted per the Declaration of Helsinki and Tokyo for humans and the current Good Clinical Practice. The protocol was approved by the Institutional Ethics Committee of Shanghai Children’s Medical Center (SCMCIRB-K2022046-1) and was registered on ClinicalTrials.gov, number NCT05501288. We reported the results of our trial following the Consolidated Standards of Reporting Trials guidelines.

### 2.1 Study medication

The Chinese Medicine composition HSBDG (Chinese medicine C20210002, batch number J2204029, specification: 5 g in each bag) was produced and provided by Guangdong Yifang Pharmaceutical Co., Ltd. Each bag is composed of Houpu (Magnoliae Officinalis Cortex), Cangzhu (Atractylodis Rhizoma), Caoguo (TsaokoFructus), Mahuang (Herba Ephedrae), Kuxingren (Armeniacae Semen Amarum), Shigao (Gypsum Fibrosum), Gancao (Glycyrrhizae Radix et Rhizoma), Huoxiang (PogostemonisHerba), Fabanxia (Pinelliae Rhizoma Praeparatum), Fuling (Poria), Shengdahuang (Rhei Radix et Rhizoma), Shenghuangqi (Astragali radix), Tinglizi (Descurainiae Semen Lepidii Semen), and Chishao (Paeoniae Radix Rubra) (Liu et al., 2021). The drug quality standards complied with the provisions of part I of the 2020 edition of the Chinese Pharmacopoeia. Compound pholcodine oral solution (HC20130002, batch number 6220021, Bright Future Pharmaceuticals Factory, Hong Kong, CHN) is composed of pholcodine, triprolidine hydrochloride, pseudoephedrine hydrochloride, and guaifenesin.

### 2.2 Participants and setting

Children who attended the COVID-19 Fangcang Shelter Hospitals in Shanghai, China, with laboratory-confirmed mild COVID-19 were screened for eligibility. The inclusion criteria were as follows: 1) compliance with the diagnostic criteria for mild COVID-19 (National Health Commission of the People’s Republic of China National Administration of Traditional Chinese Medicine, 2022); 2) age 3–18 years. Participants who had underlying disease(s) such as chronic pulmonary disease, immunodeficiency, tumors, etc., allergy or intolerance to taking Chinese medicine herbs or compound pholcodine oral solution, or poor compliance (defined as inability to comply with the protocol) were excluded.

### 2.3 Sample size

Two-sample T-tests allowing unequal variance were used for sample size calculation by Power Analysis and Sample Size Software 2021 (NCSS, LLC. Kaysville, Utah, United States of America). Group sample sizes of 64 and 32 achieve 85.0% power to reject the null hypothesis of equal means when the population mean difference is μ1 - μ2 = 10–11 = -1 with standard deviations of 1.42 for the intervention group and 1.56 for the control group (Liu et al., 2021), and with a significance level (alpha) of 0.05 using a two-sided two-sample unequal-variance *t*-test. The aim was to recruit 108 participants to allow for a 12% attrition rate.

### 2.4 Randomization and blinding

Patients were randomly assigned 2:1 to the intervention group or the control group by a researcher who was not otherwise involved in the study using random sequences generated by SPSS software 25.0 (IBM SPSS Statistics, Armonk, NY, United States of America). Three researchers applied for randomization number by smartphone and assigned participants to interventions. This was an open-label trial. Patients, investigators, and statisticians were not masked to the group assignment.

### 2.5 Intervention

In the intervention group, patients were given HSBDG the day after randomization, with a dose of 2.5 g for age 3–6 years, 5 g for age 7–12 years, and 10 g for age 13–18 years, twice daily for five consecutive days. In the control group, patients were given compound pholcodine oral solution the day after randomization, with a dose of 5 ml for age 3–6 years and 10 ml for age 7–18 years, three times daily for five consecutive days. The day of randomization was set as day 0. With free Instant messaging software (WeChat; Tencent, Shenzhen, CHN) available on smartphones, parents received questionnaires (Supplementary Table S1 for symptom scores on days 0, 3, and 5) and be asked to complete them truthfully. For each patient, one SARS-CoV-2 nucleic acid real-time PCR test for the specimen from the upper respiratory tract was performed daily. Patients would be not considered to be discharged until two consecutive negative reports were confirmed.

### 2.6 Primary outcome

The primary outcome of the study was the time for SARS-CoV-2 nucleic acid negative conversion after randomization. The time from day 0 to the day of two consecutive negative reports confirmed was defined as the negative conversion time.

### 2.7 Secondary outcomes

The secondary outcomes for the study were symptom scores on days 3 and 5, and antibiotic use and side effects from day 1 to discharge. Total symptom score was the sum of the scores of primary symptoms (fever, cough, expectoration, sore throat, wheezing, and chest pain) and secondary symptoms (dry stool, dark urine or oliguria, poor appetite, low energy, tired, nausea or vomiting, and diarrhea) (Supplementary Table S1). Based on the TCM syndromes and signs integral standard in the guideline on clinical trial design of TCM for acute upper respiratory tract infection in children (China Association of Chinese Medicine, 2022), each primary symptom was divided into four grades: no problem (score 0), minor problem (score 2), moderate problem (score 4), and major problem (score 6). Each secondary symptom was divided into four grades: no problem (score 0), minor problem (score 1), moderate problem (score 2), and major problem (score 3).

### 2.8 Statistical analysis

Data were presented as frequencies and percentages for categorical variables and as medians and interquartile range (IQR) for continuous variables. Categorical variables were compared by the chi-square test. Continuous variable comparisons were performed by the Mann-Whitney *U* test. *p*-value <0.05 was considered to be statistical significance. All analyses were performed using SPSS software 25.0 (IBM SPSS Statistics, Armonk, NY, United States of America).

## 3 Results

### 3.1 Patients

**FIGURE 1:**
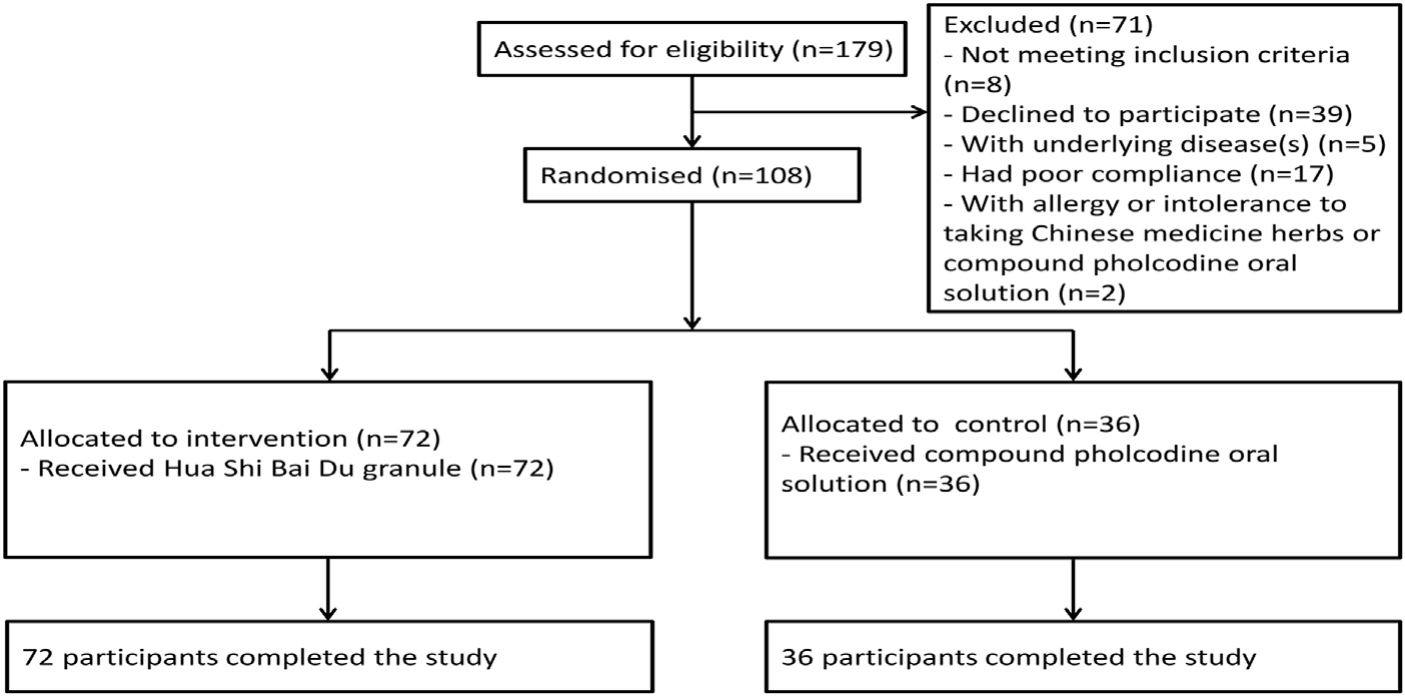
Flow diagram showing the progress of participants through the trial.

From 1 May 2022, to 30 June 2022, 179 patients with laboratory-confirmed mild COVID-19 aged 3–18 years were assessed for eligibility. After exclusions, 108 participants were enrolled and randomly assigned (72 to the intervention group and 36 to the control group) (Figure 1). Baseline characteristics were similar in both groups (Table 1). 108 children (100.0%) in total completed the study protocol. None of the participants developed moderate or severe cases.

**TABLE 1:** Characteristics of study participants at randomization.

	Intervention group (n = 72)	Control group (n = 36)	p-Value
Age, median years (IQR)	8 (5–11)	9 (7–11)	0.596
Male, No. (%)	43 (59.7)	16 (44.4)	0.133
Duration of positive SARS-CoV-2 nucleic acid detection prior to randomization, median days (IQR)	2 (1–3)	2 (1–2)	0.127
Total symptom score, median score (IQR)	3 (1–5)	5 (2–7)	0.087
Receipt of traditional Chinese medicine prior to study enrollment, no. (%)	22 (30.6)	5 (13.9)	0.059
Receipt of Western medicine prior to study enrollment, no. (%)	32 (44.4)	19 (52.8)	0.413
Vaccination, No. (%)	50 (69.4)	30 (83.3)	0.121
Weight, median kg (IQR)	37 (26–55)	36 (28–47)	0.779
Height, median cm (IQR)	138 (120–158)	140 (127–154)	0.876
Body mass index, median kg/m2 (IQR)	18.7 (15.5–22.0)	17.8 (15.2–20.0)	0.179

IQR, interquartile range.

### 3.2 Primary outcome

**FIGURE 2:**
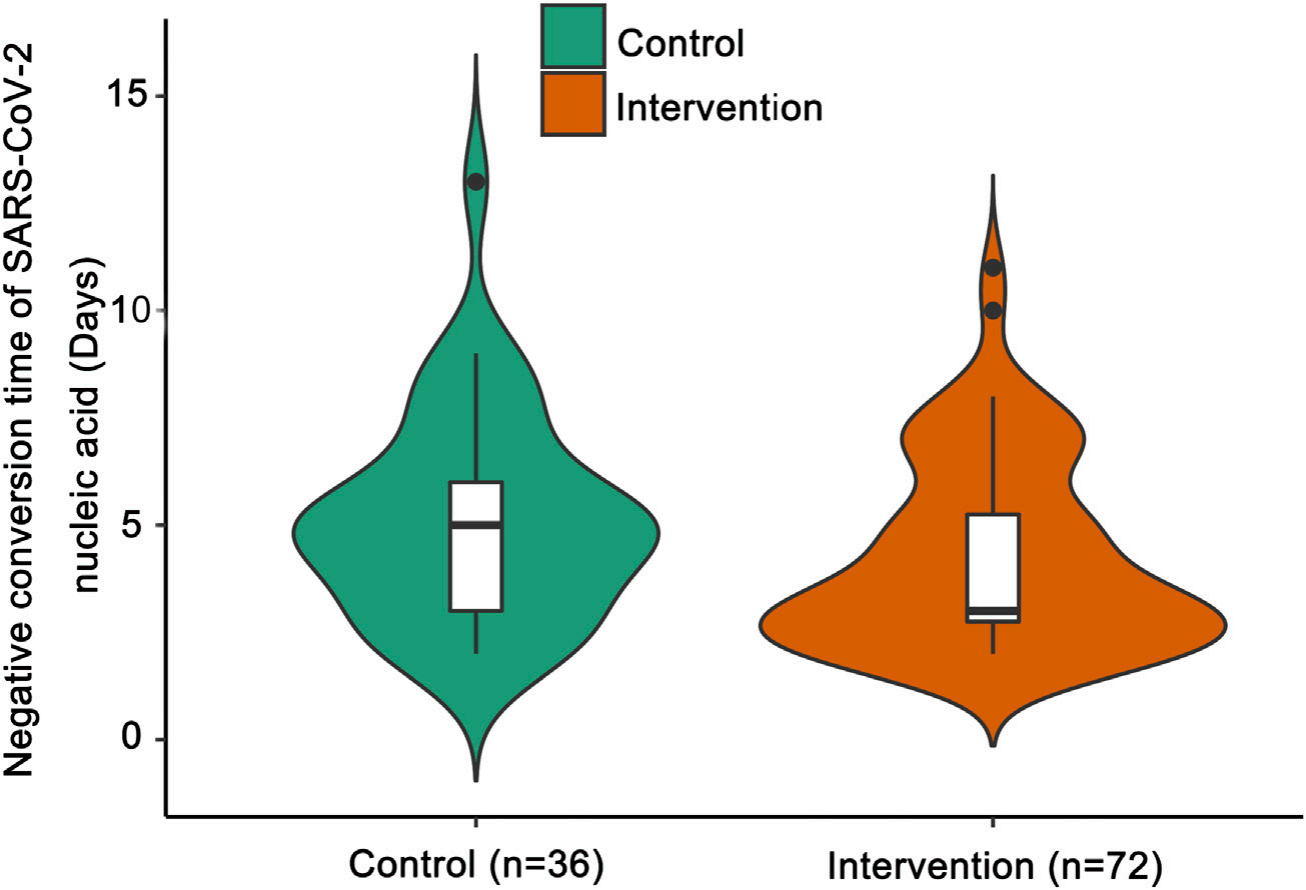
Negative conversion time of SARS-CoV-2 nucleic acid. The shapes of the violin plots represent the distributions of data. The insides of the violins are box plots. ● represents an outlier. Compared with the control group by the Mann-Whitney *U* test, the median negative conversion time of SARS-CoV-2 nucleic acid was significantly shorter in the intervention group (*p* = 0.047).

Compared with the control group, the median negative conversion time of SARS-CoV-2 nucleic acid was significantly shorter in the intervention group (median days [IQR]: 3 [3–5] vs. 5 [3–6]; *p* = 0.047; Figure 2).

### 3.3 Secondary outcome

The median total symptom score on day 3 was significantly lower in the intervention group than in the control group (median total symptom score [IQR]: 1 [0–2] vs. 2 [0–3]; *p* = 0.036). The median total symptom score on day 5 was similar between the two groups (median total symptom score [IQR]: 1 [0–2] vs. 1 [0–3]; *p* = 0.528). There was no significant difference in the frequency of antibiotic use (4.2% vs. 2.8%, *p* = 1.000) and side effects (4.2% vs. 0.0%, *p* = 0.535) between the two groups. The side effects were nausea (n = 1), vomiting (n = 1), and poor appetite (n = 1).

## 4 Discussion

We conducted a single-center, open-label, parallel-group randomized controlled trial to assess the efficacy and safety of HSBDG in pediatric patients with laboratory-confirmed mild COVID-19. Good outcomes were achieved in this trial.

HSBDG has good efficacy for different types of COVID-19, although there are differences in virus clearance. In a retrospective case series of 55 severe COVID-19 adult patients, HSBDG combined with TCM injections was observed to have good effects on SARS-CoV-2 RNA clearance, lung lesion improvement, and inflammation reduction (Wang et al., 2021). In the randomized controlled trial conducted in hospitalized adult patients with general type COVID-19, HSBDG has been proved to be efficacious and safe in improving symptoms and chest computed tomography results but not the SARS-CoV-2 nucleic acid negative conversion time (Liu et al., 2021). In our study, the first registered randomized controlled trial to evaluate the efficacy and safety of HSBDG in pediatric patients with laboratory-confirmed mild COVID-19, we proved that HSBDG could reduce the time for SARS-CoV-2 nucleic acid negative conversion besides symptom improvement.

The pharmacological effects of HSBDG may mainly involve anti-inflammation and immune regulation (Zhu et al., 2021). Most of the available data related to the potential mechanism of HSBDG is based on virtual simulation or prediction, which is acquired *via* molecular docking and network pharmacology analysis (Xia et al., 2021). HSBDF could alleviate the expression levels of IL-6 and TNF-α in the cell models (Wei et al., 2021). Network pharmacology and molecular docking analysis showed that the main active compounds of HSBDG including quercetin, ursolic acid, and rutin lead to an anti-inflammation effect by down-regulating IL-6 (Niu et al., 2021). Another network pharmacology research showed that the top two compounds of HSBDG, baicalein and quercetin, may play a therapeutic role on COVID-19 by mainly regulating TNF signaling pathway, PI3K-Akt signaling pathway, NOD-like receptor signaling pathway, MAPK signaling pathway, and HIF-1 signaling pathway through ACE2 (Tao et al., 2020). To further verify the exact mechanism of HSBDG on COVID-19, high-quality studies by molecular biological techniques *in vivo* and *in vitro* are needed.

Consistent with previous studies, the safety of HSBDG was good and no serious adverse events were reported in our study (Liu et al., 2021; Wang et al., 2021). The main side effects of HSBDG in children were gastrointestinal reactions.

Limitations existed in our study. First, due to ethical issues, we set up the compound pholcodine oral solution as a control instead of a placebo. However, no evidence shows that compound pholcodine oral solution which is used to relieve respiratory symptoms such as cough, runny nose, and sore throat has antiviral effects. Second, due to the lack of severe cases and location constraints, the efficacy and safety of HSBDG in pediatric COVID-19 patients with severe or common types were not assessed. Third, the open-label rather than double-blind design may lead to biased assessments. Fourth, as the COVID-19 Fangcang Shelter Hospitals were for medical isolation, we did not test serum cytokines for the host immune response evaluation.

## 5 Conclusion

HSBDG is capable of improving symptoms in pediatric patients with mild COVID-19. The shorter median negative conversion time of SARS-CoV-2 nucleic acid in the intervention group suggests that HSBDG may inhibit the virus replication. The exact mechanism needs further studies.

## Data Availability

The original contributions presented in the study are included in the article/Supplementary Material, further inquiries can be directed to the corresponding authors.
