# Supplementary material for: Huashi baidu granule in the treatment of pediatric patients with mild coronavirus disease 2019: A single-center, open-label, parallel-group randomized controlled clinical trial
Source: Front Pharmacol. 2023 Jan 19;14:1092748. doi: 10.3389/fphar.2023.1092748 (PMC9892187; doi:10.3389/fphar.2023.1092748)
Supplement: Supplementary file 1 [file Table1.DOCX]

Table S1. Symptom score at days 0, 3 and 5

| Primary symptom | No problem（0） | Minor problem（2） | Moderate problem（4） | Major problem（6） |
| --- | --- | --- | --- | --- |
| Fever |  |  |  |  |
| Daytime cough ^a^ |  |  |  |  |
| Nocturnal cough ^a^ |  |  |  |  |
| Expectoration |  |  |  |  |
| Sore throat |  |  |  |  |
| Wheezing |  |  |  |  |
| Chest pain |  |  |  |  |
| Secondary symptom | No problem（0） | Minor problem（1） | Moderate problem（2） | Major problem（3） |
| Dry stool |  |  |  |  |
| Dark urine or oliguria |  |  |  |  |
| Poor appetite |  |  |  |  |
| Low energy, tired |  |  |  |  |
| Nausea or vomiting |  |  |  |  |
| Diarrhea |  |  |  |  |

^a^ Cough score is the average of daytime cough score and nocturnal cough score.
